# Supplementary material for: Enhancing Protoplast Isolation and Early Cell Division from Cannabis sativa Callus Cultures via Phenylpropanoid Inhibition
Source: Plants (Basel). 2024 Jan 2;13(1):130. doi: 10.3390/plants13010130 (PMC10781014; doi:10.3390/plants13010130)
Supplement: Supplementary file 1 [file plants-13-00130-s001.zip › plants-2666046-supplementary.pdf]

Supplementary Materials to:

Communication

Enhancing Protoplast Isolation and Early Cell Division from *Cannabis sativa* Callus Cultures via Phenylpropanoid Inhibition

Adrian S. Monthony [1,2,3,4] and Andrew Maxwell P. Jones[5*]

[1]Département de phytologie, Université Laval, Québec City, Québec, Canada
[2]Institut de Biologie Intégrative et des Systèmes (IBIS), Université Laval, Québec, Canada
[3]Centre de recherche et d'innovation sur les végétaux (CRIV), Université Laval, Québec, Canada
[4]Institut intelligence et données (IID), Université Laval, Québec, Canada
[5]Department of Plant Agriculture, University of Guelph, Guelph, Canada
* Correspondence: amjones@uoguelph.ca

Monthony ORCID: 0000-0003-0536-691X
*Jones ORCID: 0000-0001-9106-5341

Supplementary Methods

Protoplast Isolation and Culture

Callus tissues from each treatment were prepared as in the Materials and Methods of the manuscript. Each digestion was conducted in a 10 mm Petri dish (VWR International) using 15 mL of enzyme solution per 300 mg of callus tissue. After a 16-hour digestion, protoplast purification followed the method outlined by Beard *et al.* [1] with slight modifications as outlined below.

Petri dishes were examined under an inverted microscope (Axiovert 200) to confirm digestion and check for contamination. The enzyme solution was then filtered twice using a 70 μm and a 40 μm cell strainer in a sterile laminar flow hood. The filtrate was transferred to a screw cap tube and centrifuged at 1000 RPM (~178 G) for 10 minutes in a hanging bucket centrifuge (IEC HN-SII General Purpose Centrifuge, Needham Heights, MA, USA). The supernatant was removed, and the pellet was resuspended in 3 mL of a matrix solution.

Protoplast purification was performed using a 60% (w/v) iodixanol (OptiPrep, Sigma-Aldrich, 1.32 g/mL) density gradient (Figure S1A). The protoplast suspension was mixed with a 40% OptiPrep working solution (WS; 0.4 M mannitol in OptiPrep) in a 3:2 ratio until homogenous. This mixture was then layered over a 20% OptiPrep solution (0.6 mL OptiPrep WS + 2.4 mL matrix solution) in a screw cap tube. The tube was centrifuged at 200 G for 10 minutes. Following centrifugation, the protoplast band at the interface between the 20% and 0% OptiPrep layer (Figure S1A) was carefully collected using a wide-mouthed micropipette tip and the volume of the band removed was recorded (V_{band}). A 30 μL aliquot was sampled for protoplast counting using a hemocytometer under a compound light microscope to determine their concentration (a). The total number of protoplasts (b_{total}) from the band was used to determine the protoplast yield (y_{proto}), measured in protoplasts per gram of fresh callus weight (w_{fresh}) as shown in Eqs. S1 and S2.

$$b_{total} = a \times V_{band} \quad \text{(Eq. S1)}$$

$$y_{proto} = b_{total} \times w_{fresh} \quad \text{(Eq. S2)}$$

A 100 μL aliquot was stained for cell viability with 2 mg/mL fluorescein diacetate (FDA; Sigma-Aldrich) at a ratio of 60 μL FDA per mL of sample and incubated in the dark for 10 minutes. Viability was assessed by counting the ratio of live protoplasts (based on fluorescence) to total protoplasts in three randomly selected locations on a prepared slide, using an inverted epifluorescence microscope equipped with a FITC/Bodipy/Fluo 3/DiO Filter Set (Chroma, Bellows Falls, VT, USA).

The protoplast band was then suspended in an equal volume of KM5/5 media comprised of Kao and Michayluk (KM) salts and vitamins [2] containing 10 % (w/v) mannitol, 1% (w/v) sucrose, 500 mg/L MES, 5 μM BA and 5 μM NAA adjusted to a pH of 5.7 and centrifuged for 10 minutes at 178G. The supernatant was removed, and the protoplast pellet was

resuspended in a volume equal to twice the target culture density using the KM5/5 media. This suspension was mixed with an equal volume of 1.6% (w/v) low melting point SeaPlaque agarose solution (Mandel Scientific, Guelph, ON, Canada) maintained at 38 °C to achieve the desired protoplast density. The mixture was transferred dropwise to a 6-well tissue culture-grade polystyrene plate with one drop (~100 µL) in each well. Subsequently, 1.5 mL of sterile KM5/5 media (with or without 10 µM AIP) was added to each well after the agarose beads had solidified (~20 minutes). The cultures (Figure S1B) were kept in the dark in a controlled environment growth chamber at 25 °C and regularly monitored for cell division. Various protoplast concentrations in the low melting point agarose beads were tested, ranging from 0.5×105 to 2.0×105 protoplasts/mL.

Total Phenols and Browning Assay

Total phenols in callus were determined using a modified Folin-Ciocalteu (F-C) assay with a gallic acid (Sigma-Aldrich, St. Louis, MO) standard curve [3–5]. Each analysis day began with the preparation of a gallic acid stock solution (2 mg/mL) in extraction buffer, from which a 7-point standard curve in the range of 50 to 1000 µg/mL was established (Figure S2A). The analysis closely followed the method by Jones and Saxena [3], with minor adjustments. In a 96-well flat-bottomed microplate (C3370, Corning Inc., Corning, NY, USA), 100 µL of F-C Reagent (MP Biomedicals, Santa Ana, CA, USA) was added to each well, followed by a 10 µL aliquot of the extract. Wells with extraction buffer alone served as blank controls. Similar wells with varying concentrations of gallic acid standard were also prepared. Lastly, 80 µL of aqueous 0.25 M Na_2CO_3 was added to each well, with careful removal of any bubbles under gentle vacuum. The microplate was then incubated in the dark at 30 °C for 1 hour using a microplate spectrophotometer (Epoch 2, BioTek, Winooski, VT, USA). Following incubation, the plate underwent 20 seconds of orbital mixing, and the absorbance was measured at 765 nm [4].

Absorbance at 340 nm was measured as a proxy for tissue browning as previously reported in Jones and Saxena [3]. Ferulic acid (Sigma-Aldrich) was used as a standard to estimate the total phenolic content of the previously isolated extracts. Each analysis day began with the preparation of a ferulic acid stock solution (2 mg/mL) in extraction buffer, from which a 7-point standard curve in the range of 50 to 1000 µg/mL was established (Figure S2B). Standard curves were run in triplicate and distributed evenly across the microplate, with a new standard curve prepared and run with each microplate. In a 96-well plate (Corning Inc.), 190 µL of extraction buffer was added to each well, followed by 10 µL aliquots of sample extracts, standards, or sample blanks. Eleven sample blanks were included on each plate, distributed throughout to address intra-plate variation. The plate underwent 20 seconds of orbital mixing, and absorbances at 340 nm of the samples were measured at 30 °C using a microplate spectrophotometer (BioTek). The standard curves demonstrated strong linearity (R^2>0.99) for each standard curve between 50 µg/mL and 1000 µg/mL in both assays (Figure S2, **Table S1** and **Table S2**)

Supplementary Figures

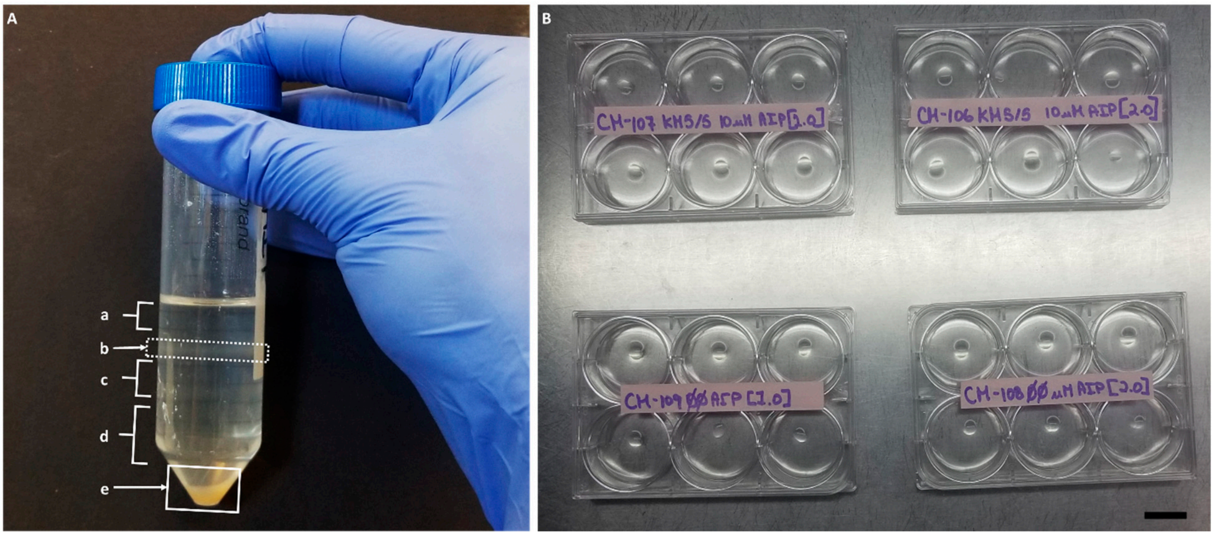

Figure S1. Protoplast purification and culture system. A) OptiPrep density gradient post-centrifugation. a) Top matrix solution layer (0% OptiPrep). b) Protoplast accumulation at the 0%-20% OptiPrep interface (collected for downstream applications and culture). c) The 20% OptiPrep gradient. d) The 40% OptiPrep solution containing initial protoplast suspension post-digestion, with cell debris at the tube bottom (e). B) Protoplasts in low melting point agarose beads in 1.5 mL KM5/5 media with 0 µM and 10 µM AIP.

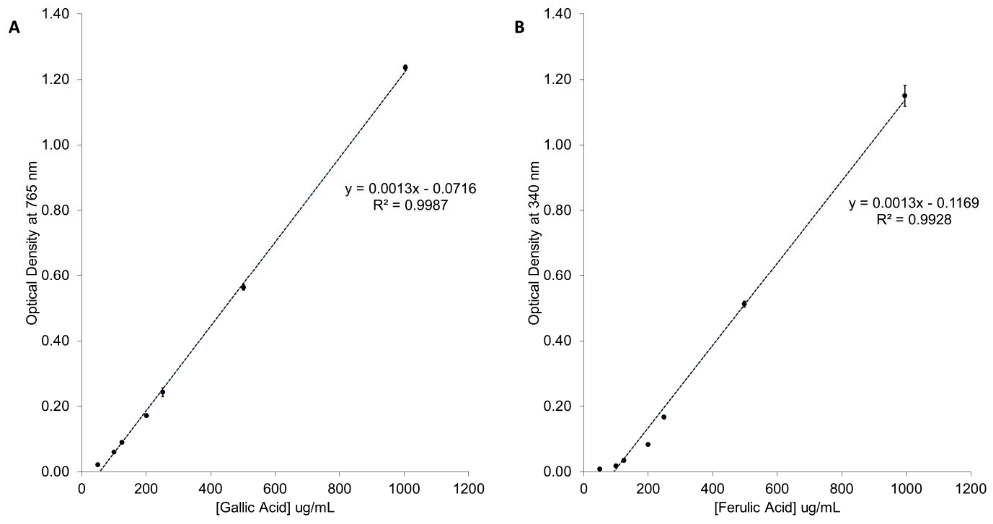

Figure S2. Representative standard curves obtained for the F-C assay (A) and for the browning assay (B). Both curves show strong linearity (R^2>0.99). Each data point represents the mean of 3 readings (n=3), error bars indicated standard error of the mean. Data was processed and graphs prepared using Microsoft Excel™.

Supplemental Tables

Table S1. Daily F-C assay calibration curves concentrations, coefficient of variation and linear regression equations. Calculated using Microsoft Excel™. GAE: gallic acid equivalent. Calibration Curves were obtained between May 03, 2021 and May 07, 2021.

Daily Concentration Range (µg GAE /mL)	R^2	Linear Regression Equation
50.2-1004	0.9983	y=0.0014x-0.0409
50.5-1010	0.9993	y=0.0014x-0.0539
50.4-1008	0.9982	y=0.0012x-0.0443
50.2-1004	0.9987	y=0.0013x-0.0716

Table S2. Daily browning assay calibration curves concentrations, coefficient of determination (R^2) and linear regression equations. Calculated using Microsoft Excel™. FAE: ferulic acid equivalents. Calibration Curves were obtained between May 03, 2021 and May 07, 2021.

Daily Concentration Range (µg FAE/mL)	R^2	Linear Regression Equation
50.7-1014	0.9942	y=0.0012x-0.1128
50.8-1016	0.9953	y=0.0013x-0.1091
50.5-1010	0.9911	y=0.0013x-0.1270
49.8-996	0.9928	y=0.0013x-0.1169

Table S3. Results of the F-test from the ANOVA of the protoplast yield. Yield (protoplasts/gram fresh weight) obtained from enzymatically digested *Cannabis sativa* callus. α=0.05.

Fixed Effects	Numerator df	Denominator df	F Value	P-value
Media	1	36	5.07	0.0305
Subculture Frequency	1	36	0.00	0.9852
Media×Subculture Frequency	1	36	2.31	0.1369

Table S4. Results of the F-test from the ANOVA of the F-C assay. Assay quantifies total soluble phenolics (µg GAE/gram fresh weight) obtained from *Cannabis sativa* callus. α=0.05.

Fixed Effects	Numerator df	Denominator df	F Value	P-value
Media	1	34	8.87	0.0053
Subculture Frequency	1	34	0.14	0.7092
Media×Subculture Frequency	1	34	0.95	0.3375

Table S5. Results of the F-test from the ANOVA of the browning assay. Assay quantifies browning measured as ferulic acid equivalents (µg FAE/gram) obtained from *Cannabis sativa* callus. α=0.05.

Fixed Effects	Numerator df	Denominator df	F Value	P-value
Media	1	17.69	47.88	<0.0001
Subculture Frequency	1	17.69	0.01	0.9208
Media×Subculture Frequency	1	17.69	0.50	0.4887

Table S6. Kendall Tau-b correlation table. This table displays Kendall Tau-b correlation coefficients, significance levels (α=0.05) for the test of null hypothesis (no correlation), and the number of observations used for ranked correlation out of a total of n=40. Some data points were omitted due to limited sample extract availability caused by insufficient callus for extract preparation.

	Yield	F-C	Browning
	1.000	-0.20827	-0.24480
**Yield**	-	0.692	0.0322
	40	38	38
	-0.20827	1.000	0.37237
**F-C**	0.692	-	0.0012
	38	38	37
	-0.24480	0.37237	1.000
**Browning**	0.0322	0.0012	-
	38	37	38

Supplementary Materials References

1. Beard, K.M.; Boling, A.W.H.; Bargmann, B.O.R. Protoplast isolation , transient transformation , and flow-cytometric analysis of reporter-gene activation in *Cannabis sativa* L. *Ind. Crop. Prod.* **2021**, *164*, 1–7, doi:10.1016/j.indcrop.2021.113360.
2. Kao, K.N.; Michayluk, M.R. Nutritional requirements for growth of Vicia hajastana cells and protoplasts at a very low population density in liquid media. *Planta* **1975**, *126*, 105–110, doi:10.1007/BF00380613.
3. Jones, A.M.P.; Saxena, P.K. Inhibition of phenylpropanoid biosynthesis in Artemisia annua L.: A novel approach to reduce oxidative browning in plant tissue culture. *PLoS One* **2013**, *8*, 1–13, doi:10.1371/journal.pone.0076802.
4. Kupina, S.; Fields, C.; Roman, M.C.; Brunelle, S.L. Determination of total phenolic content using the Folin-C assay: Single-laboratory validation, first action 2017.13. *J. AOAC Int.* **2019**, *102*, 320–321, doi:10.5740/jaoacint.2017.13.
5. Singleton, V.L.; Orthofer, R.; Lamuela-Raventós, R.M. [14] Analysis of total phenols and other oxidation substrates and antioxidants by means of folin-ciocalteu reagent. In *Methods*; 1999; Vol. 299, pp. 152–178.
